# Do Perceived Values Influence User Identification and Attitudinal Loyalty in Social Robots? The Mediating Role of Active Involvement

**DOI:** 10.3390/bs15101329

**Published:** 2025-09-28

**Authors:** Hua Pang, Zhen Wang, Lei Wang

**Affiliations:** 1 School of New Media and Communication, Tianjin University, Tianjin 300072, China; 2 Department of Linguistics, University of Konstanz, 78464 Konstanz, Germany

**Keywords:** perceived values, user identification, attitudinal loyalty, social robot

## Abstract

With the rapid advancement of artificial intelligence, the deployment of social robots has significantly broadened, extending into diverse fields such as education, medical services, and business. Despite this expansive growth, there remains a notable scarcity of empirical research addressing the underlying psychological mechanisms that influence human–robot interactions. To address this critical research gap, the present study proposes and empirically tests a theoretical model designed to elucidate how users’ multi-dimensional perceived values of social robots influence their attitudinal responses and outcomes. Based on questionnaire data from 569 social robot users, the study reveals that users’ perceived utilitarian value, emotional value, and hedonic value all exert significant positive effects on active involvement, thereby fostering their identification and reinforcing attitudinal loyalty. Among these dimensions, emotional value emerged as the strongest predictor, underscoring the pivotal role of emotional orientation in cultivating lasting human–robot relationships. Furthermore, the findings highlight the critical mediating function of active involvement in linking perceived value to users’ psychological sense of belonging, thereby elucidating the mechanism through which perceived value enhances engagement and promotes sustained long-term interaction. These findings extend the conceptual boundaries of human–machine interaction, offer a theoretical foundation for future explorations of user psychological mechanisms, and inform strategic design approaches centered on emotional interaction and user-oriented experiences, providing practical guidance for optimizing social robot design in applications.

## 1. Introduction

Social robots are autonomous agents designed to engage with humans in socially appropriate manners, integrating advances in language processing, computer vision, and affective computing to enable diverse functionalities (Abadie et al., 2024; H. Kim et al., 2022; Giger et al., 2025). Driven by sophisticated algorithms and market demand, social robots have become deeply embedded in the interactive networks of the digital society, gradually integrating into daily life through advanced technologies. Given their substantial capacity for development, these technologies have demonstrated broad applicability across key sectors, notably education, healthcare, and business (Liu et al., 2024; Lin & Wu, 2023). By including both companion robots and socially assistive robots, this study enables a more comprehensive examination of human–robot interactions and a deeper understanding of user perceptions and behaviors. The ongoing integration of social robots into social contexts highlights their importance, while also presenting challenges related to maintaining user engagement, satisfaction, and loyalty, all of which are essential for ensuring their continued effectiveness. Given these challenges, this study introduces the concept of perceived value to explore how perceived value connects these factors with the mechanisms that promote users’ continuous intention to use social robots Broadly speaking, perceived value encapsulates the benefits and overall value derived from the interaction between users and social robots. It represents a multidimensional construct encompassing utilitarian value, emotional value, and hedonic value (Hong et al., 2025; Suphasomboon & Vassanadumrongdee, 2022; H. Yang et al., 2016). These dimensions collectively shape users’ evaluations and decisions regarding the continued use of social robots, emphasizing their pivotal role in influencing behavioral intentions.

At present, although a growing body of empirical research has examined perceived value in human–computer interaction (Hong et al., 2025; Shahzad et al., 2024; Y. Hu, 2021), important limitations remain in the context of social robots. First, most studies on the adoption of social robots have emphasized their anthropomorphic features, such as voice, gestures, or facial expressions, and patterns of human–robot interaction (Gao et al., 2025; Yuan et al., 2024). While such perspectives are valuable, they often overlook the integration of broader theoretical frameworks, which restricts a more comprehensive understanding of the psychological mechanisms shaping user behavior. Second, many empirical investigations pay insufficient attention to the mediating role of active user involvement. Few have systematically analyzed how social robots can stimulate engagement behaviors, such as users’ willingness to interact, explore functions, or sustain long-term use (Chen et al., 2025; Al-Abdullatif & Alsubaie, 2024). Third, the majority of current literature remains confined to surface-level analyses of technological features and user experience, with limited efforts to explore deeper psychological constructs, including user identification and attitudinal loyalty. Some studies have examined the role of user participation (Goyal & Verma, 2024; Lin & Wu, 2023), yet gaps remain in understanding how short-term engagement evolves into more enduring psychological commitments. This gap raises the critical question of how weak or situational connections between users and machines can be transformed into stable forms of loyalty, which also limits our understanding of the broader social implications of social robots.

Building on prior research, this study examines perceived value in social robotics (Hong et al., 2025; Li & Shang, 2020) to capture how users evaluate their interactions with social robots. By integrating theoretical insights with empirical investigation, the study highlights the combined effects of these value dimensions and examines how active involvement as a mediating mechanism through which users translate perceptions of value into both behavioral responses and emotional commitment. In contrast to prior research that has primarily focused on identifying the determinants of users’ behavioral acceptance of social robot technologies (Gong, 2025; Zhu et al., 2023), this study extends the scope of analysis to deeper psychological and behavioral outcomes by incorporating user identification and attitudinal loyalty. In doing so, it not only broadens the concept of loyalty to users’ loyalty and identification with non-human entities but also enriches its application within AI and social robotics. In addition, the study highlights the critical influence of active involvement on engagement, identification, and loyalty, offering practical implications for social robot design and interaction strategies. These contributions help establish a more comprehensive behavioral model for social robot users and offer guidance for both academic inquiry and industry practice. The specific research hypotheses derived from this framework are presented in the following sections.

## 2. Theoretical Framework and Hypotheses Development

### 2.1. Linking Perceived Values to Active Involvement

In general, perceived values encapsulate a consumer’s integrated evaluation of a product’s functional worth, influenced by their individual cognitive and emotional responses (Hong et al., 2025; Al-Abdullatif & Alsubaie, 2024; Suphasomboon & Vassanadumrongdee, 2022). This evaluation is subjective and multidimensional, reflecting both cognitive factors, such as functionality, quality, and cost-effectiveness, and emotional experiences, such as enjoyment and satisfaction (Mpinganjira et al., 2024; Peng et al., 2019; H. Yang et al., 2016). The present research suggests that, beyond serving practical purposes, social robots foster emotional interaction and enjoyment through tailored communication and entertainment content. Consequently, perceived value is conceptualized in three key dimensions: utilitarian, emotional, and hedonic (Hong et al., 2025; Al-Abdullatif & Alsubaie, 2024), with particular attention to the mediating function of users’ active involvement. It is widely accepted that involvement constitutes an individual’s subjective appraisal of relevance, shaped by internal needs, values, and interests (Molinillo et al., 2022). In this study, users’ active involvement with social robots is defined as their proactive and sustained engagement with social robots, encompassing emotional resonance, cognitive appraisal, and behavioral involvement. Specifically, utilitarian value refers to the perceived benefits derived from the functionality, usability, expected quality, and performance of a product (Hong et al., 2025; Suphasomboon & Vassanadumrongdee, 2022). Emotional value primarily reflects the specific emotional responses and feelings that a product evokes in an individual (Suphasomboon & Vassanadumrongdee, 2022; Peng et al., 2019). Meanwhile, hedonic value draws on the concept of hedonism, represents the sensory gratification and enjoyment gained through consumption and related activities, such as fun and entertainment (Fang et al., 2025; El-Adly, 2019).

Studies on perceived values span various domains, including consumer behavior, branding and marketing, digital technologies and related disciplines (Mpinganjira et al., 2024; Wang et al., 2021). Previous studies have shown that emotional experiences, service quality, and cost factors influence perceived value, which in turn affects satisfaction and behavioral intentions (Al-Abdullatif & Alsubaie, 2024; Wang et al., 2021). With advancements in digital technology, research on perceived value has increasingly focused on how products such as online platforms and smart devices provide customized services that enhance users’ perceived values. In social commerce environments, the reliability of information and services is a major determinant of customers’ perceived value, which subsequently influences their loyalty behavior (Molinillo et al., 2021). Moreover, recent research has demonstrated that the perceived utilitarian value of AI chatbots serves as a mediating factor linking interactivity, information quality, and individual trust to the purchase intentions of potential tourists, thereby informing this study (Zhu et al., 2023).

Specifically in the field of social robots, these technologies shape users’ perceived value through their functional attributes and the relationships they cultivate with users, thereby influencing user behavior (Y. Hu, 2021; Hong et al., 2025; Shahzad et al., 2024). Perceived values, including information seeking, social interaction, and entertainment satisfaction, increase user engagement and likelihood of continued use (Mpinganjira et al., 2024; Lin & Wu, 2023; Gong, 2025). Additionally, recent research indicates that users’ perceptions of intelligence and high performance in social robots can significantly enhance their participation and intention to engage with these technologies (Fang et al., 2025; Lin & Wu, 2023). Consequently, taken together, users’ perceived values of social robots significantly influence interaction quality, involvement, loyalty, and continuous use intention. Drawing on the aforementioned research background, the study puts forward the following hypotheses:

**H1.**  *Utilitarian value is positively associated with active involvement.*

**H2.**  *Emotional value is positively associated with active involvement.*

**H3.**  *Hedonic value is positively associated with active involvement.*

### 2.2. Linking Active Involvement to Attitudinal Loyalty and User Identification

Loyalty and identification represent two critical psychological constructs in user research (Gong, 2025; Dikcius et al., 2024; Kaur et al., 2020). Loyalty, conceptualized as a cognitive-affective attitude, reflects users’ tendencies to engage in behaviors driven by positive evaluations of the loyalty target (Dikcius et al., 2024; Chiu et al., 2013). Loyalty can be categorized into attitudinal loyalty and behavioral loyalty (Wolter et al., 2017), with this study mainly focusing on attitudinal loyalty. In this context, attitudinal loyalty is defined as a positive and enduring disposition of users towards social robots, manifested by users’ strong willingness to maintain interaction with the robots and recommend them to others. Identification, initially defined as an individual’s self-understanding and self-positioning (Divya & Christopher, 2024), has evolved to include diverse forms such as organizational, brand community, and social media influencer identification (Kaur et al., 2020; Wolter et al., 2017; White & Yu, 2024). In the digital realm, individuals increasingly develop a sense of identification, foster emotional intimacy, and form relational connections through their interactions with virtual platforms and technological agents such as social robots (Gong, 2025; Lin & Wu, 2023). Specifically, users’ identification with social robots embodies a multidimensional connection encompassing cognitive, emotional, and behavioral aspects, which are forged through these ongoing interactions (Chen et al., 2025).

Prior studies examining active involvement as a factor in cultivating user attitudinal loyalty and identification predominantly centers on consumer behavior, particularly within the realms of brand marketing and relationship marketing (Kaur et al., 2020; Gummerus et al., 2012; Fang et al., 2025). Empirical studies have emphasized that active involvement positively influences attitudinal loyalty, as demonstrated by user behaviors (Kamboj & Rahman, 2016) and their usage patterns on online booking platforms (Goyal & Verma, 2024). Similarly, co-creation of shared experiences via involvement, communication, and empathy has been found to strengthen user identification within live streaming platforms (M. Hu et al., 2017). Furthermore, research indicates that positive user experiences significantly bolster electronic brand loyalty by encouraging active interaction, thereby strengthening loyalty toward brands offering chatbot service (Gomes et al., 2025; Shahzad et al., 2024). Although existing research predominantly addresses brand communities and digital service platforms, the mechanism of active involvement in the field of human–robot interaction has received comparatively limited attention.

Social robots, characterized by their capacity for social presence and perceived agency, introduce a unique interactive setting that blends technological utility with emotional engagement (Gong, 2025; Yuan et al., 2024; Fang et al., 2025). Active involvement in such contexts can intensify relational dynamics and deepen user-robot interactions, thereby laying the foundation for loyalty and identification (Liu et al., 2024). Moreover, heightened involvement enhances the perception of anthropomorphism, facilitating the development of rapport between users and chatbots and thereby positively influencing word-of-mouth communication and further strengthens emotional bonds and relational ties (Kayeser Fatima et al., 2024). Given the above analysis, this study argues that users’ active involvement with social robots not only reflects a positive evaluation of the abilities and benefits of social robots, but also lays the groundwork for a deeper emotional connection, leading to user identification and attitudinal loyalty with social robots. In other words, active involvement serves as a crucial predictor for fostering both attitudinal loyalty and user identification. Consequently, the following two hypotheses are proposed in this study:

**H4.**  *Active involvement is positively associated with attitudinal loyalty.*

**H5.**  *Active involvement is positively associated with user identification.*

### 2.3. Linking User Identification to Attitudinal Loyalty

The association between user identification and attitudinal loyalty has been a major focus of research guided by social identity theory (Kaur et al., 2020; M. Hu et al., 2017; He et al., 2012). According to this theory, individuals develop a sense of belonging to a group through a process of self-categorization or self-classification (Stets & Burke, 2000). Identification occurs when individuals perceive that a particular group or entity embodies important aspects of their own identity and fulfills self-definitional or self-expressive needs. In such instances, the identified object gains psychological significance, fostering a deeper sense of personal alignment and belonging (Wolter et al., 2017). When individuals develop a positive sense of satisfaction and identification, their relationship with the brand, organization, community, or technology tends to become more enduring, giving rise to emotional commitment and attachment (Shukla et al., 2023; Chang et al., 2020). This identification-driven commitment reflects the individual’s loyal attitude toward the identified object (Divya & Christopher, 2024; Kaur et al., 2020). Empirical research indicates that identification with a community, brand, or organization not only strengthens users’ sense of belonging and emotional engagement but also establishes a robust and enduring foundation for formation of attitudinal loyalty. This identification facilitates sustained support and proactive advocacy, thereby facilitating sustained support (White & Yu, 2024; Confente & Kucharska, 2021).

Given the expanding role of social robots within daily activities, exploring how users develop identification with these interactive technologies is essential to understanding their long-term loyalty and engagement. Identification promotes attitudinal loyalty by fostering emotional commitment, enabling users to perceive the robot as more than a utilitarian tool, but as an emotionally significant partner (Yuan et al., 2024; Leo-Liu, 2023). Moreover, the personalized interactions provided by social robots play a pivotal role in shaping user identification. These interactions allow users to perceive the robot’s human-like qualities, along with tailored services and emotional support, fostering a positive identification (Gong, 2025). This identification, together with the development of trust and user satisfaction, encourages users to maintain ongoing interactions with social robots, reflecting a high level of attitudinal loyalty (Chen et al., 2025; Shahzad et al., 2024). This attitudinal loyalty manifests specifically in their consistent utilization of social robots, their propensity to offer favorable evaluations, and their willingness to actively recommend these robots to others (Hong et al., 2025). Accordingly, it is reasonable to state that user identification serves as a pivotal factor in fostering attitudinal loyalty among users. Based on the above arguments, this study proposes the following hypothesis:

**H6.**  *User identification is positively associated with attitudinal loyalty.*

## 3. Research Methodology

### 3.1. Research Model

**Figure 1:**
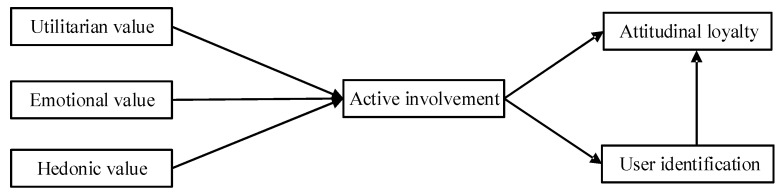
The conceptual research model.

Figure 1 presents the research model employed in this study, outlining the hypothesized relationships among the core variables. The analysis is conceptually grounded in perceived value theory (H. Yang et al., 2016), providing the theoretical basis for exploring user evaluation and engagement. Within this framework, utilitarian, emotional, and hedonic values are hypothesized to have positive effects on users’ active involvement with social robots. Active involvement, in turn, is expected to positively influence both user identification and attitudinal loyalty. Additionally, user identification is proposed as a further antecedent of attitudinal loyalty, thereby reinforcing its formation and stability.

### 3.2. Sample and Data Collection

The empirical data were obtained through Sojump (www.sojump.com), a professional online survey platform in China that provides access to a broad and diverse participant pool. By leveraging this platform, the study achieved wide demographic coverage and improved sample representativeness, while also benefiting from the platform’s recognized methodological strengths in delivering data with superior reliability, efficiency, and scalability compared with conventional survey techniques (Pang et al., 2024). To ensure adherence to the principles of voluntariness and anonymity, all participants were fully informed in advance about the study’s purpose and procedures. The data collection period spanned from 27 January 2025 to 7 March 2025. Ultimately, a total of 614 participants completed the questionnaire. Following the exclusion of cases lacking prior interaction, with implausibly short response times, or containing inconsistent information, 569 valid questionnaires remained. These validated questionnaires formed the basis for subsequent empirical testing and analysis.

### 3.3. Measurement

The questionnaire employed in this study comprises two primary parts. The first part covers demographic information such as gender, age, educational attainment, previous experience with social robots, and average daily interaction time. All demographic variables were categorized following established research practices and the observed data distribution to ensure meaningful analysis. The second part focuses on four key constructs: perceived value, active involvement, user identification, and attitudinal loyalty, which have been elaborated in the preceding hypothesis development. To measure perceived value, this study builds upon the foundational work of C. Kim et al. (2012) and Peng et al. (2019), which established validated scales for utilitarian, emotional, and hedonic value. Specifically, these dimensions are highly pertinent for examining users’ perceptions of social robots with respect to functional services, informational exchange, and affective support. In addition, to capture active involvement, this study draws on the measurement scale developed by K. Yang et al. (2015), originally designed for user participation in social shopping websites. This instrument is especially suitable because it captures both motivational and behavioral aspects of user engagement. Finally, in measuring user identification and attitudinal loyalty, the research refers to the variable designs proposed by M. Hu et al. (2017) and Wolter et al. (2017), respectively, while incorporating novel adaptations tailored to the context of social robots. More concretely, M. Hu et al. (2017) introduced a dual identification framework in the context of live video streaming platforms, providing robust measures of users’ sense of identification with a medium or technology. Wolter et al. (2017), in the retailing context, advanced the measurement of attitudinal loyalty by emphasizing users’ affective commitment, relational attachment, and loyalty conviction. Building on these established contributions, the present study adapts and extends their scales to the context of social robots, thereby maintaining theoretical rigor and contextual relevance. The specific measurement items and their sources are detailed in Table 1. Collectively, these constructs provide a comprehensive framework for analyzing user perceptions and behaviors in social robot interactions.

## 4. Data Analysis Strategy

This study adopted a quantitative research methodology, collecting data via questionnaires and analyzing them using SPSS 27.0 and AMOS 29.0. Data preprocessing involved removing invalid samples based on response time and logical consistency. To control for common method bias (CMB), Harman’s single-factor test was conducted. The data were also examined for outliers and missing values, with consistency tests performed to ensure data integrity. Descriptive statistical analyses were performed using SPSS 27.0 to characterize variable distributions. Cronbach’s alpha (>0.7) was used to evaluate the internal consistency reliability of the scale, while confirmatory factor analysis (CFA) using AMOS 29.0 assessed the structural validity of the measurement model. Reliability was further confirmed through standardized factor loadings (>0.5) and the composite reliability index (CR > 0.7). Convergent validity was evaluated based on the average variance extracted (AVE > 0.5) to verify psychometric adequacy. Multicollinearity among the predictors was examined using variance inflation factors (VIF) and tolerance values, following commonly recommended thresholds (Gong, 2025; Shahzad et al., 2024). All variables satisfied these criteria, indicating that multicollinearity did not threaten the stability or interpretability of the regression coefficients. Subsequently, a latent variable path model was constructed and its fit assessed via multiple indices. Theoretical hypotheses were tested using standardized path coefficients (>0.20) and significance levels (*p* < 0.05), with indirect effects examined through bias-corrected bootstrap confidence intervals to identify potential mediating pathways.

## 5. Results

### 5.1. Demographic Statistics

Before exploring the proposed hypothesis, a demographic analysis of the sample was performed in this study to obtain underlying descriptive statistics. The research cohort comprised 569 participants, with gender distribution documented as 301 males (52.9%) and 268 females (47.1%). Results showed that the age distribution of respondents revealed a predominance of younger participants, with the majority falling within the 19–29 years old range 44.82%. This was closely followed by individuals aged 30–40 years old 39.02%. The average age of participants was about 29.1 (SD ≈ 8.4). Educational attainment data indicated 41.5% of respondents held undergraduate degrees, followed by 32.2% senior high school students, 14.1% with junior high school education or below. Regarding social robot engagement duration, the majority of respondents (35.15%) reported engagement with social robots for 1–2 years, while 27.42% had 2–4 years of experience. Most participants (34.80%) indicated spending 1–2 h daily social robot use, closely followed by roughly 30% engaging for 2–4 h. Overall, the sample composition aligns well with the target population of this study, with participants demonstrating substantial experience in social robot usage, thereby ensuring the relevance and generalizability of the findings. Potential demographic effects on perceived value were examined, and no significant differences were found, indicating that the main results are robust across demographic groups. Table 2 presents a general overview of the demographic profiles of the valid participants.

### 5.2. Measurement Model, Reliability, and Validity

In this study, the fit of the model and the quality of the measurement tool were tested by multi-dimensional indexes. To test the reliability and validity of all measures used in this research, we performed a confirmatory factor analysis (CFA).

In terms of model evaluation, the results indicated a satisfactory overall fit. The absolute fit indices, including the chi-square divided by degrees of freedom (χ^2^/df = 1.572), the root mean square error of approximation (RMSEA = 0.032), and the root mean square residual (RMR = 0.042), were all within the recommended thresholds. Likewise, the incremental fit indices, namely the comparative fit index (CFI = 0.983), the adjusted goodness-of-fit index (AGFI = 0.931), the incremental fit index (IFI = 0.983), and the Tucker–Lewis index (TLI = 0.981), further confirmed that the proposed model demonstrated an excellent fit to the data. In the evaluation of structural equation model fit, although chi-square statistic was sensitive to small model differences in a large sample scenario, taking into account other robust fit indicators and sample size, the data still support the overall fit between the model and the observed variables. This conclusion shows that it does not substantially weaken the statistical validity and theoretical explanatory power of the model. Table 3 presents the detailed model fit indices, with all key metrics meeting or exceeding established psychometric thresholds, thereby confirming the model’s validity and alignment with statistical standards.

Measurement reliability was evaluated using Cronbach’s alpha and composite reliability, both of which exceeded the accepted threshold of 0.70 (Lin & Wu, 2023), confirming the model’s internal consistency. Regarding convergent validity, confirmatory factor analysis (CFA) results showed that all standardized factor loadings surpassed 0.70. In addition, the average variance extracted (AVE) and square multiple correlations (SMC) for all constructs were above the 0.50 threshold, supporting strong convergent validity. Discriminant validity results are presented in Table 4. The measurement model exhibited robust psychometric properties. As shown in Table 5, the square root of the AVE for each construct exceeded its correlations with other constructs, confirming discriminant validity (Fornell & Larcker, 1981). These results collectively indicate satisfactory model fit, high reliability, and acceptable convergent validity for the measurement instruments used in this study.

### 5.3. Common Method Bias

The research employed standardized survey instruments for data collection, a methodology that potentially introduces CMB. In order to avoid the interference of unsystematic bias of CMB, the study adopted a strict anonymity policy, requiring respondents to remain neutral and honest, and to respond according to their own actual situation (Shahzad et al., 2024). To ensure the robustness of data quality, this study performed Harman’s single-factor test and applied exploratory factor analysis (EFA) to all questionnaire items, aiming to examine whether a single dominant factor accounted for the majority of the covariance among variables (Zhu et al., 2023; Gong, 2025). The analysis results show that the variance explained by the first principal component is lower than 50%, indicating that CMB was not a major concern in this survey. In addition, VIF values were assessed for all latent constructs, and all results were below the recommended threshold (Shahzad et al., 2024), confirming that multicollinearity did not substantially threaten the validity of the findings.

### 5.4. Structural Model

**Figure 2:**
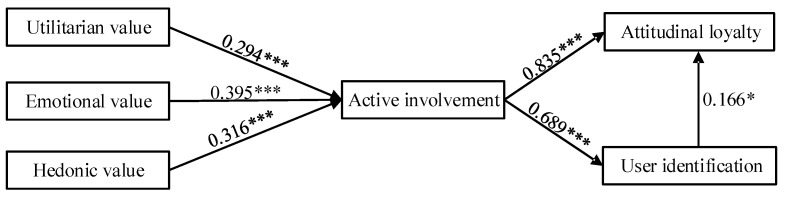
Results for the structural equation model. * *p* < 0.05; *** *p* < 0.001.

To validate the proposed framework, structural equation modeling was conducted using the empirical data. The measurement model results indicate that all fitting indexes reach the ideal level (χ^2^/d.f = 1.572 < 3; RMSEA = 0.032 < 0.08; RMR = 0.042 < 0.05; CFI = 0.983 > 0.9; AGFI = 0.931 > 0.8; IFI = 0.983 > 0.9; TLI = 0.981 > 0.9), showing that the hypothesized measurement model exhibits superior goodness-of-fit with the observed data. Subsequent analysis of the structural model was conducted to verify the hypothesized paths among latent variables. The path analysis results indicated that perceived value significantly influenced the level of active involvement. Utilitarian value (β = 0.294, *p* < 0.001), emotional value (β = 0.395, *p* < 0.001) and hedonic value (β = 0.316, *p* < 0.001) all positively predicted active involvement; thus the Hypothesis 1, Hypothesis 2, and Hypothesis 3 are supported. Active involvement substantially enhanced both user identification (β = 0.835, *p* < 0.001) and attitudinal loyalty (β = 0.689, *p* < 0.001), thereby corroborating Hypothesis 4 and Hypothesis 5. Furthermore, user identification exhibited a statistically discernible yet modest predictive capacity over attitudinal loyalty (β = 0.166, *p* = 0.023), lending empirical validation to Hypothesis 6. Therefore, the six hypotheses proposed in this study are verified and supported at conventional significance thresholds (*p* < 0.05), with five path coefficients surpassing the more stringent *p* < 0.001 criterion. The standardized path coefficients among variables are shown in Figure 2.

### 5.5. Mediation Analysis

The indirect effects of perceived value dimensions on user identification and attitudinal loyalty via active involvement were further examined, and the results are summarized in Table 6. Following the examination of the structural model, to further explore the underlying psychological mechanisms, a bias-corrected bootstrap method (N = 5000) was employed to examine the indirect effects. The results indicate that utilitarian value (β = 0.068, SE = 0.027, 95% CI [0.025, 0.134]), emotional value (β = 0.090, SE = 0.027, 95% CI [0.044, 0.150]), and hedonic value (β = 0.075, SE = 0.026, 95% CI [0.032, 0.135]) all exert significant indirect effects on user identification through active involvement. Similarly, these three value dimensions also indirectly influence attitudinal loyalty through the mediating role of active involvement, with utilitarian value (β = 0.158, SE = 0.037, 95% CI [0.091, 0.247]), emotional value (β = 0.199, SE = 0.043, 95% CI [0.123, 0.292]), and hedonic value (β = 0.162, SE = 0.036, 95% CI [0.099, 0.239]) exhibiting statistically significant indirect effects. These results confirm the robust mediating role of active involvement between perceived value dimensions and both user identification and attitudinal loyalty.

## 6. Discussion

### 6.1. Summary of the Key Findings

This empirical study proposes a hypothetical model, which mainly discusses the multidimensional perceived value of users to social robots, and depicts its correlation with user identification and attitudinal loyalty through empirical methods. It also demonstrates the prominent mediating role of active involvement in this process, showing that perceived value influences the frequency and intensity of interaction between users and social robots, which in turn affects the level of user identification and loyalty. Specifically, the study hypothesizes that utilitarian value, emotional value, and hedonic value positively influence active involvement, which subsequently enhances user identification and attitudinal loyalty. These findings offer valuable perspectives on the psychological mechanisms, further enriching the understanding of users’ willingness to interact and the underlying factors influencing human–robot interaction.

Firstly, Hypothesis 1, Hypothesis 2 and Hypothesis 3 propose that higher perceived utilitarian value, emotional value, and hedonic value lead to more positive human–computer interaction behaviors with social robots, which is consistent with existing research in the academic field (Li & Shang, 2020; Kasilingam, 2020; Fang et al., 2025). This study verifies that various aspects of perceived value all further affect users’ identification construction and attitudinal loyalty through mediating effects. Existing studies have fully demonstrated the reinforcing mechanism of multi-dimensional perceived value on users’ behavioral intentions in the field of brand marketing (Peng et al., 2019), and this theoretical framework has found further support within human–computer interaction studies. Previous research has shown that users’ perception of chatbots’ functional utility and emotional gratification positively correlates with enhanced usage satisfaction and behavioral engagement (Ashfaq et al., 2020). Building on this evidence, the current study tests these mechanisms in the context of social robotics. The findings indicate that emotional value predicts user identification and attitudinal loyalty more strongly than utilitarian or hedonic value, emphasizing the regulatory function of emotional connection in human–computer interaction. This result indicates that in human–computer interaction situations, the emotional connection mechanism plays a more core regulatory role. When users experience strong emotional value from interactions with social robots, they are inclined to establish affective ties defined by trust, attachment, and loyalty. Personalized responses and behavior that align with user preferences further enhance emotional resonance, deepening identification and strengthening relational bonds (Gong, 2025). These findings not only provide empirical support for an emotion-centered model of human–robot relationship maintenance but also suggest a promising avenue for theoretical development in social robotics.

Secondly, the establishment of Hypothesis 4 and Hypothesis 5 verified that higher levels of engagement with social robots strengthen users’ identification and foster attitudinal loyalty. Specifically, frequent interaction facilitates the formation of trust and perceived reliability, enabling users to view robots as credible and valuable interaction partners. This research result indicates that there is a dual-path effect in this influence mechanism. On the one hand, when users interact more with social robots, this high-frequency interaction can establish a sense of trust and reliability through a continuous cognitive reinforcement process (Shahzad et al., 2024), facilitate users’ identity construction toward social robots, fostering their perception of robots as trustworthy interaction partners. On the other hand, the literature establishes that implementing familiar relational paradigms in human–computer interaction substantially increases user acceptance rates (Yuan et al., 2024), and this effect is particularly pronounced in high-engagement contexts, where such interaction patterns have been shown to foster authentic, enduring user loyalty (Goyal & Verma, 2024). Building on these insights, sustained engagement emerges as a dynamic process in which emotional attachment accumulates over time, underpinning stable attitudinal loyalty and continued preference for the same robot. Such a perspective offers a process-oriented explanatory framework for understanding the long-term maintenance of human–robot relational dynamics.

Thirdly, the empirical validation of Hypothesis 6 confirms that user identification significantly influences attitudinal loyalty, which corresponds with findings documented in the wider body of research (Chen et al., 2025). This study holds that stimulating users’ feeling of attachment and identity is conducive to generating a more lasting sense of loyalty than simply enhancing the satisfaction with the functions of social robots. Furthermore, while the positive association between user identification and attitudinal loyalty has been statistically confirmed, the relatively small effect size suggests the relationship may also be moderated by other unexplored factors. Research findings reveal that the anthropomorphic characteristics of social robots shape users’ responses and their attitudes during interactions (Gomes et al., 2025). The age and gender of individuals have different focuses in their perception of value and also affect the level of loyalty to a certain extent (Kamboj & Rahman, 2016). In contexts involving more sophisticated and specialized applications of social robots, anthropomorphic design features exert significant multidimensional effects on user engagement, loyalty and usage intentions (Leo-Liu, 2023; Liu et al., 2024). Taken together, the findings suggest that optimizing social robot design requires the integrated consideration of functional utility, emotional connectivity, and interactive entertainment, with particular attention to delivering emotional value that enhances users’ engagement and participation, thereby reinforcing identification, loyalty, and long-term usage intentions.

Ultimately, the mediation analysis results highlight the pivotal role of active involvement as a psychological mechanism linking perceived value to user identification and attitudinal loyalty toward social robots. Specifically, active involvement functions as a significant mediator, transmitting the influence of utilitarian value, emotional value, and hedonic value on both identification and loyalty outcomes. This result is consistent with previous studies highlighting the crucial influence of user participation levels on individual behavior and decision-making in digital and human–machine interaction settings (Peng et al., 2019; Goyal & Verma, 2024). As such, active involvement emerges as a key psychological variable for understanding users’ sense of identification and behavioral tendencies, offering a deeper explanation of how affective and cognitive evaluations evolve into lasting attitudinal commitments (Shukla et al., 2023).These findings indicate that user loyalty toward social robots is shaped not only by perceived utilitarian, emotional, and hedonic value, but also by the extent of users’ active involvement. Such engagement enhances both the frequency and depth of interaction, thereby reinforcing user identification and fostering more favorable attitudinal responses. In sum, the mediation results provide a more nuanced understanding of how perceived value translates into user identification and attitudinal loyalty, emphasizing active involvement as a pivotal conduit in this relationship and offering actionable insights for advancing human–robot interaction research and practice.

### 6.2. Theoretical and Practical Implications

This research holds substantial theoretical and practical significance. From a theoretical perspective, firstly, it extends the analytical lens beyond traditional behavioral models to provide a deeper understanding of human–robot interaction. While prior researches predominantly center on surface-level behavioral constructs such as the Technology Acceptance Model and usage intention (Gong, 2025; Gao et al., 2025), this study introduces the multi-dimensional concept of perceived value, encompassing functional, emotional, and hedonic dimensions, to highlight its role in shaping users’ internal psychological mechanisms. This emphasis on internal cognitive and affective processes distinguishes our work from existing studies that largely focus on observable usage behaviors. Secondly, this study develops an integrated framework that connects perceived value, active involvement, user identification, and attitudinal loyalty. By explicitly focusing on users’ active engagement with social robots, it demonstrates how overall evaluations of these technologies foster psychological belonging, self-relevance, and sustained interaction intentions. This approach differs from prior studies that treat engagement as a secondary or indirect factor, highlighting its pivotal role in the emergence of robot-specific loyalty. Thirdly, this study combines multidisciplinary theories and attempts to integrate perceived value theory, identity theory and other theoretical contents in theoretical integration to form a systematic interpretation framework (Kaur et al., 2020; H. Yang et al., 2016), which provides a reusable measurement tool and analysis framework for subsequent research. This kind of theoretical integration not only improves the theoretical depth of the research, but also provides theoretical support and analytical framework for the subsequent study of human–robot relationship from the perspectives of psychology, society and technology. Finally, this study investigates social robots as independent interactive objects, rather than symbol carriers attached to brands or enterprises. Although previous research on human–robot relationships has recognized the importance of user loyalty, it has predominantly emphasized loyalty toward the companies or brands behind the robots, rather than considering the robots themselves as autonomous agents of interaction (Shahzad et al., 2024; Liu et al., 2024). This distinction suggests that loyalty mechanisms in human–robot interaction may operate differently from traditional brand loyalty, depending more on immediate emotional engagement and active participation rather than mediation through brand identity or corporate affiliation. This shift in perspective contributes to the theoretical advancement of human–robot relationship research by moving beyond brand-oriented loyalty toward the emerging concept of robot loyalty. It further extends the conceptual boundaries of human–machine interaction, provides a theoretical foundation for future research on user psychological mechanisms, and offers a paradigmatic reference for the interdisciplinary development of human–robot interaction studies.

This study also carries significant practical implications, which are primarily reflected in the following aspects. Firstly, this study offers a user-centered foundation for product optimization, providing valuable insights for social robot designers and service providers aiming to enhance functionality, emotional appeal, and user experience. Empirical research and analysis show that every dimension of perceived value has a differentiated impact mechanism on user participation, identification and loyalty, which provides support for enterprises to accurately locate design priorities. For example, according to the relative influence weights of different dimensions, enterprises can prioritize the emotional value dimension, optimize the personalized and customized emotional interaction functions of social robots to develop design strategies with more psychological insight. Secondly, by deconstructing the multi-dimensional pathway of perceived value, this study offers methodological guidance for enterprises seeking to move beyond a purely functionalist approach and establish design frameworks centered on users’ psychological mechanisms. With the rapid development and application of social robots in daily companionship and life services, users’ long-term willingness to use social robots has become the key to enhancing competitiveness. By revealing the internal formation mechanism of user attitudinal loyalty, this study finds that active involvement is an important factor affecting the long-term attitude maintenance of users. This also provides a behavioral pathway reference for user engagement and relationship maintenance strategies. Prior research has demonstrated that anthropomorphic features can facilitate effective user interaction, foster harmonious human–robot relationships, and enhance user participation (Kayeser Fatima et al., 2024). Therefore, enterprises can develop more accurate user relationship maintenance strategies, such as enhancing the level of active participation of users by strengthening the personality and personalized interaction level of social robots, so as to enhance user stickiness and willingness to use. Finally, from the perspective of user psychological construction, this study offers strategic insights for enterprises aiming to develop differentiated competitive advantages centered on emotion first and identity driven principles. The findings reveal that user identification with social robots significantly enhances attitudinal loyalty. Importantly, this identification is not solely rooted in the robots’ functional capabilities, but also emerges from a deeper emotional and value-based resonance among users, robots, and brands. When users feel valued and connected, their need for relevance will be satisfied (White & Yu, 2024). Meanwhile, more engaged users tend to show stronger willingness to continue interaction and brand loyalty (Kaur et al., 2020). Therefore, enterprises can address users’ emotional, cognitive, and social needs to foster a sense of belonging and perceived fit, thereby strengthening the psychological connection between users and social robots and enhancing the core competitiveness of social robotics products. In essence, this study not only bridges the existing gap in the research on user identification and attitudinal loyalty of social robots in theory, but also offers a practical path for enterprises to build user-centered competitive advantages and realize the transformation from technology-oriented to user-demand-oriented design paradigm in the increasingly fierce human–computer interaction market.

## 7. Limitations and Implications for Future Research

Despite offering substantial academic and practical contributions, this study is not without limitations. Firstly, the quantified research data is representative to a certain extent, but the proportion of participants with higher education in this study is slightly over half, which may also have sampling bias or digital divide effect, making it difficult to describe the psychological state of the full range of social robot users completely and objectively. Secondly, the current findings primarily elucidate general patterns in user-robot interaction, while acknowledging significant variations across service typologies and functional domains. Specifically, the mechanisms underlying user identification and loyalty formation may differ substantially between application contexts such as healthcare and education, requiring domain-specific investigations to uncover the moderating factors. Thirdly, limited by the short-term interaction data collected, the findings may not fully reflect the dynamic development of users’ attitudes and behavioral patterns in sustained human–robot relationships. Future research can employ longitudinal designs to systematically track the dynamic progression of these interactions over extended periods. Finally, the study acknowledges that beyond identification, other potential factors influencing user attitudinal loyalty were not examined within the scope of this research. Subsequent studies could propose a more detailed and integrative model to better capture the complexity of these influences.

## Figures and Tables

**Table 1:** Measurement and questionnaire.

Variable	Item	Source
Utilitarian value	(1) I tend to interact with the social robot when I have something specific I need to inquire or request. (2) When interacting with the social robot, I tend to seek only information or services that I need/want. (3) The social robot enables convenient access to information or services. (4) The social robot provides the information and services I need.	(C. Kim et al., 2012)
Emotional value	(1) Social robots can totally absorb me. (2) I enjoy the feeling of interacting with the social robot. (3) Interacting with social robots and obtaining services is a pleasant way. (4) Using social robots makes me feel quite happy.	(Peng et al., 2019)
Hedonic value	(1) For me, social robots can provide me with entertainment experiences. (2) It is pleasant and fun to interact with social robots. (3) I get excited when I obtain information and services from social robots. (4) I lose track of time when I interact with and obtain information and services from social robots.	(C. Kim et al., 2012)
Active involvement	(1) I’m motivated to actively interact with social robots. (2) I interact frequently with social robots. (3) I’m excited to message with social robots and get replies. (4) I often obtain information and services from social robots.	(K. Yang et al., 2015)
User identification	(1) I intend to recommend the social robot that I regularly use to people around me. (2) I intend to continue to use the social robot that I regularly use. (3) Social bots can convey values or message content that is important to me. (4) The information and services provided by social robots are similar to my views and needs.	(M. Hu et al., 2017)
Attitudinal loyalty	(1) The relationship that I have with the social robot is something I am very committed to. (2) I really care about my ongoing relationship with the social robot. (3) I consider my relationship with the social robot worth keeping. (4) I think the social robot is the superior choice.	(Wolter et al., 2017)

**Table 2:** Summary of demographic information (N = 569).

Categories	Frequency	Percentage (%)
Gender		
Males	301	52.90%
Females	268	47.10%
Age		
≤18 years old	50	8.79%
19–29 years old	255	44.82%
30–40 years old	222	39.02%
≥41 years old	42	7.38%
Educational background		
Junior high school or below	80	14.06%
Senior high school	183	32.16%
Undergraduate degree	236	41.48%
Doctoral degree or above	70	12.30%
Engagement time with social robots		
Less than 1 year	136	23.90%
1–2 years	200	35.15%
2–4 years	156	27.42%
More than 4 years	77	13.53%
Time spent on social robots daily		
Less than 1 h	135	23.73%
1–2 h	198	34.8%
2–4 h	166	29.17%
More than 4 h	70	12.3%

**Table 3:** Fit indices for the measurement model.

Model Fit Measures	Model Fit Criterion	Index Value	Good Model ft (Y/N)
Absolute ft indices			
RMSEA	<0.08	0.032	Y
RMR	<0.05	0.042	Y
χ^2^/d.f. (χ^2^ = 418.023, d.f. = 266)	<3	1.572	Y
Incremental ft indices			
CFI	>0.90	0.983	Y
AGFI	>0.80	0.931	Y
IFI	>0.90	0.983	Y
TLI	>0.90	0.981	Y

**Table 4:** Statistical results of confirmatory factor analysis.

Constructs and Items	Loading (>0.7)	SMC (>0.5)	CR (>0.7)	AVE (>0.5)
Utilitarian value (UV)			0.887	0.611
UV1	0.795	0.632		
UV2	0.790	0.625		
UV3	0.766	0.587		
UV4	0.760	0.577		
UV5	0.796	0.633		
Emotional value (EV)			0.876	0.638
EV1	0.796	0.633		
EV2	0.808	0.653		
EV3	0.810	0.655		
EV4	0.781	0.610		
Hedonic value (HV)			0.870	0.626
HV1	0.800	0.640		
HV2	0.803	0.644		
HV3	0.789	0.623		
HV4	0.773	0.598		
Active involvement (AI)			0.873	0.632
AI1	0.806	0.649		
AI2	0.776	0.602		
AI3	0.791	0.625		
AI4	0.807	0.651		
User identification (UI)			0.877	0.640
UI1	0.788	0.621		
UI2	0.818	0.669		
UI3	0.804	0.647		
UI4	0.792	0.628		
Attitudinal loyalty (AL)			0.868	0.621
AL1	0.781	0.610		
AL2	0.791	0.626		
AL3	0.825	0.680		
AL4	0.754	0.568		

Notes: SMC, squared multiple correlations; CR, construct reliability; AVE, average variance extracted.

**Table 5:** Discriminant validity.

	UV	EV	HV	AI	UI	AL
UV	**0.884**					
EV	0.634 ***	**0.894**				
HV	0.622 ***	0.661 ***	**0.890**			
AI	0.683 ***	0.727 ***	0.703 ***	**0.892**		
UI	0.717 ***	0.746 ***	0.715 ***	0.761 ***	**0.894**	
AL	0.647 ***	0.719 ***	0.674 ***	0.776 ***	0.741 ***	**0.888**

Notes: *** *p* < 0.001. UV, Utilitarian value; EV, Emotional value; HV, Hedonic value; AI, Active involvement; UI, user identification; AL, attitudinal loyalty. Diagonal elements (bold) represent AVE. Off-diagonal elements represent squared correlations between variables.

**Table 6:** Analysis of the mediation effect.

Path	Effect	β	SE	Lower	Upper	*p*
UV → AI → UI	Indirect effect	0.068	0.027	0.025	0.134	***
EV → AI → UI	Indirect effect	0.090	0.027	0.044	0.150	***
HV → AI → UI	Indirect effect	0.075	0.026	0.032	0.135	***
UV → AI → AL	Indirect effect	0.158	0.037	0.091	0.247	***
EV → AI → AL	Indirect effect	0.199	0.043	0.123	0.292	***
HV → AI → AL	Indirect effect	0.162	0.036	0.099	0.239	***

Notes: *** *p* < 0.001. UV, Utilitarian value; EV, Emotional value; HV, Hedonic value; AI, Active involvement; UI, user identification; AL, attitudinal loyalty.

## Data Availability

The original contributions presented in this study are included in the article. Further inquiries can be directed to the corresponding authors.
